# Supplementary material for: Integrated model for Food-Energy-Water (FEW) nexus to study global sustainability: The water compartments and water stress analysis
Source: PLoS One. 2022 May 13;17(5):e0266554. doi: 10.1371/journal.pone.0266554 (PMC9106181; doi:10.1371/journal.pone.0266554)
Supplement: S1 File — Section S1: Sectoral water intensity equations are presented in this section. Section S2: This section describes the method adopted for modelling regions in this work. (PDF) [file pone.0266554.s001.pdf]

Integrated model for Food-Energy-Water (FEW) nexus to study global sustainability: The water compartments and water stress analysis

Neeraj Hanumante[1☯], Yogendra Shastri[1☯], Apoorva Nisal[2‡], Urmila Diwekar[2, 3‡], Heriberto Cabezas[4‡]

1 Department of Chemical Engineering, Indian Institute of Technology Bombay, Mumbai, Maharashtra, India

2 Department of Industrial Engineering, University of Illinois, Chicago, IL, USA

3 Vishwamitra Research Institute, Crystal Lake, IL, USA

4 Research Institute for Applied Earth Sciences, University of Miskolc, Miskolc, Hungary

☯These authors contributed equally to this work.

‡These authors also contributed equally to this work.

* Corresponding author

Email: urmila@vri-custom.org

S1 Sectoral water intensity equations

Sectoral intensity trends and their equations are shown in Figure S1 and S2

S2 Modelling regions

The water demand distribution is computed as shown in Figure S3.

It is elaborated as shown below:

1. Identify the shadow variables for each sector on country level and obtain data for these shadow variables:

   The identified shadow variables are shown in Table S1:

2. Convert per capita data to total values:

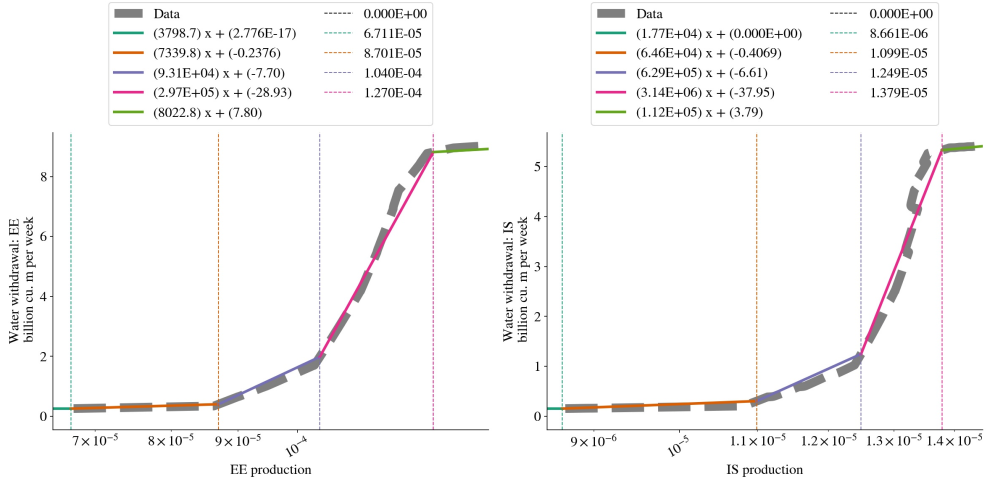

Fig S1. Sectoral intensity trends: Industrial and Energy sector

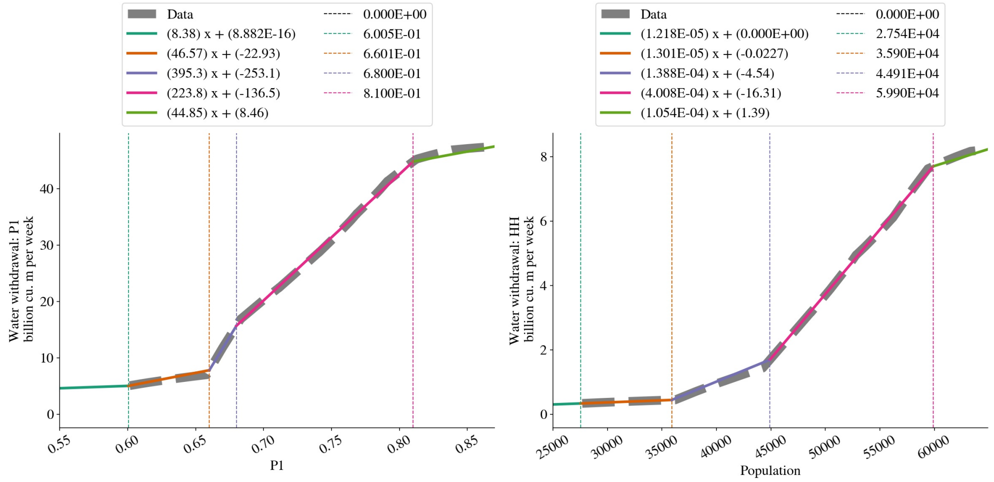

Fig S2. Sectoral intensity trends: Agricultural and Municipal sector

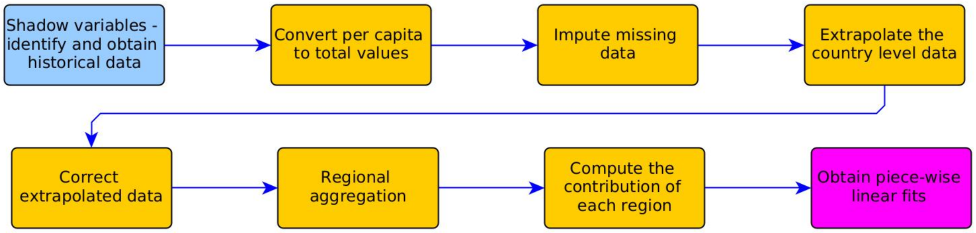

Fig S3. Steps in modelling regions

Table S1. Shadow variables for different sectors

Agriculture	Agricultural area	[1]	Hectares per capita
Livestock	Meat production	[2]	Tonne
Industry and Energy	GDP	[3]	GDP per capita international $
Municipal	Population	[4]	Number of persons

From Table S1, the agricultural land and GDP are per capita variables. These are converted using the corresponding population to total values.

3. Impute missing data:

   Linear interpolation is used for imputation of the missing data. Backward linear extrapolation is used to obtain the data from 1950 to 1961.

4. Extrapolate the country level data:

   Data from 1992 to 2013 is used to extrapolate .

5. Correction of extrapolated data:

   It should be ensured that the agricultural area is less than total area of the country and agricultural area cannot be reduced to zero. For this purpose, for each of the regions minimum ratio of agricultural area to total area is obtained. It acts as lower bound. Ratio of 1 is set as upper bound. The data is corrected based on these bounds.

6. Aggregation according to regions/groups:

   The corrected data is then aggregated based on the region they belong to. Aggregation is carried out through summation.

7. Compute contribution of each region to total values.

8. Use piece-wise linear fits for these contribution trends.

Acknowledgments

This is a collaborative project between the USA, India, and Hungary. The authors would like to thank R. Boumans for sharing data from the Global Unified Model of the BiOsphere (GUMBO) and his invaluable inputs from the same.

References

1. Our World in Data. Agricultural land use; 2021. `https://ourworldindata.org/grapher/agricultural-land?stackMode=absolute&time=1961&region=World`.

2. Ritchie H, Roser M. Meat and Dairy Production. Our World in Data. 2017;.

3. Roser M. Economic Growth. Our World in Data. 2013;.

4. Max Roser HR, Ortiz-Ospina E. World Population Growth. Our World in Data. 2013;.
