# Supplementary material for: Integrated model for Food-Energy-Water (FEW) nexus to study global sustainability: The water compartments and water stress analysis
Source: PLoS One. 2022 May 13;17(5):e0266554. doi: 10.1371/journal.pone.0266554 (PMC9106181; doi:10.1371/journal.pone.0266554)
Supplement: Attachment — Submitted filename: responsetoeditorfinal.pdf [file pone.0266554.s002.pdf]

Dear Editor,

Thank you for your quick review of our papers. I am providing the rebuttal with this letter. We are also submitting the revised manuscript where we have taken out the funding section from the acknowledgment and added the link for accessing the code. The funding format you have provided looks good, we slightly modified it as follows.

Thanks, and looking forward to your response.

Regards

Urmila Diwekar

FUNDING PARAGRAPH:

NH, YS, UD acknowledge the support from the Ministry of Human Resource Development, Government of India, through the SPARC (project code: P1238).
The research contribution by H. Cabezas was carried out in the GINOP-2.3.2-15-2016-00010 framework Development of enhanced engineering methods with the aim at utilization of subterranean energy resources" project at the Research Institute of Applied Earth Sciences of the University of Miskolc, partially funded by the European Union, co-financed by the European Structural and Investment Funds.

The funders had no role in study design, data collection and analysis, decision to publish, or preparation of the manuscript."

REBUTTAL:

Editor's comment: My first concern is that the work you present here is an extension to another paper already submitted to our journal. You must clarify what the need for a separate paper for an extension to the same model is. Indeed, this is something that it had been better to explain to the journal office during the submission to try to have the same Editor for both works. Also, both manuscripts seem to share part of the authors.
Therefore, please, justify the reason to split both works. In the meantime, I will contact the office to discuss this issue.

Response: The focus of the first paper was to describe the global sustainability model in terms of the food web and the microeconomic model. The focus of the first paper was to identify model parameters so as to align model output with historical global values. The model was then used to perform simulations for the next 100 years and study global sustainability in terms of the population of various ecological compartments, economic metrics such as GDP, and track various emissions like GHG and NOx.

The focus of the second paper, in contrast, is to study global water sustainability. Although the model is based on the one presented in the first paper, it incorporates several modifications as highlighted here:

- Water compartments as well as the stocks and flows of water are incorporated.
- The parameter values for the various equations governing the utilization of water by various sectors such as industry and agriculture are estimated using historical data.
- Since water stress is highly region specific, the model is further modified to consider different continents. The water availability, consumption, and resulting stress are, therefore, calculated and simulated for each of the continent separately, thereby providing more granular insight. Note that the model in the first paper did not study continent wise trends.

Therefore, the approach we have taken in the second paper is different from the first paper. Further, the details involved are crucial, and combining both papers would have caused more confusion. Therefore, we have split the papers. The papers can be judged separately or together. We would appreciate it if the same reviewers judged them.

Editor's Comment: Secondly, from the current title of the manuscript, it seems that you present a new model. But you do not include any code for it (at least I have not found it after a quick read). In the list of Supporting Information, you mention the equations of the model. Probably you have not computed your results by hand, but a computer implementation of them. You have to include it with the text. The same applies to the outputs of the model, the simulations that you mention. All this information is necessary to ensure the reproducibility of your work by reviewers and potential readers. Also, what would be the point of presenting a new model and limiting its use by others?

Response: We have a large code for this model which we could not submit. However, we are providing a link to google drive where the model currently sits. This drive has all the data, manual, and code. We have now included this in the current manuscript.
