# Supplementary material for: Integrated model for Food-Energy-Water (FEW) nexus to study global sustainability: The water compartments and water stress analysis
Source: PLoS One. 2022 May 13;17(5):e0266554. doi: 10.1371/journal.pone.0266554 (PMC9106181; doi:10.1371/journal.pone.0266554)
Supplement: Attachment — Submitted filename: response.pdf [file pone.0266554.s003.pdf]

Response to review comments: Integrated model for Food-Energy-Water (FEW) nexus to study global sustainability: The water compartments and water stress analysis

Neeraj Hanumante[1◑], Yogendra Shastri[1◑], Apoorva Nisal[2‡], Urmila Diwekar[2, 3‡], Heriberto Cabezas[4‡]

1 Department of Chemical Engineering, Indian Institute of Technology Bombay, Mumbai, Maharashtra, India

2 Department of Industrial Engineering, University of Illinois, Chicago, IL, USA

3 Vishwamitra Research Institute, Crystal Lake, IL, USA

4 Research Institute for Applied Earth Sciences, University of Miskolc, Miskolc, Hungary

◑These authors contributed equally to this work.

‡These authors also contributed equally to this work.

* Corresponding author

Email: urmila@vri-custom.org

Code available at:

https://github.com/arnisal/Generalized-Global-Sustainability-Model

Reviewer 1

Comment: This is a well-written paper addressing a topical subject of the WEF nexus. The authors have provided every detail including the modelling code. The authors need to add references to some of the statements in the introduction and check the grammar. But otherwise this is a very good study.

Response: Thank you for your valuable review comment. To address the concerns, we have altered the introduction to incorporate missing references and have carried out another grammar check. Relevant text is reproduced here:

> **line 5**
>
> Energy is an important contributor to human development and well-being, and it is also a key input to agriculture [1].
>
> **line 12**
>
> Additionally, excessive water withdrawal due to cheap energy may further deplete groundwater resources [2].
>
> **line 48**
>
> Currently, prima facie, the available water is adequate to match human consumption on an aggregate level [3].
>
> **line 64**
>
> Lastly, the SDG 6 measures are expected to improve the overall water management [4].

Other significant changes

To address comments of Reviewer 3, model description equations have been revised and tables summarizing parameter values and variable description are included.

Reviewer 2

Comment: This paper uses and expands the global “integrated cross-disciplinary model” known as Global Sustainability Model (GGSM) to investigate water sustainability under different future scenario of water demand. The authors claim that this is done while considering the food-water-energy nexus. The development of models of the food-water-energy nexus is a hot topic with potentially significant societal impacts. That said, I believe that the authors have not provided enough details to understand what exactly GGSM simulates and how. For example:

How are the different water resources allocated within each country?

Response: We thank the reviewer for the comment.

We would like to clarify that the modeling of water resources within each country is not carried out in the current study.

The aim of this work is to investigate the long-term regional demand-supply dynamics for various sectors and hence, analyzing the continent-level water stress (line 55). The GGSM is a global model and hence, in order to explore the regional effect of water stress, the global water system is disaggregated into six continents. The allocation of water resources in these continents is modelled based on FAO, UN data [5] (line 243). However, internal allocation within the continent is not modelled and hence, modelling intra-national allocation of water resources is beyond the scope of the current study.

Comment: How are “economic and technological considerations” taken into account to assess how much water can be harvested?

Response: The “economic and technological considerations” are taken into account by modelling the water reservoir in terms of exploitable water resources. Exploitable water is defined by AQUASTAT as follows (line 105 in the manuscript):

> “Exploitable water resources (also called manageable water resources or water development potential) are considered to be available for development,

> taking into consideration factors such as: the economic and environmental feasibility of storing floodwater behind dams, extracting groundwater, the physical possibility of storing water that naturally flows out to the sea, and minimum flow requirements (navigation, environmental services, aquatic life, etc). Methods to assess exploitable water resources vary from country to country."

Comment: How is water demand of the different sector satisfied?

Response: The water demand by different sectors is satisfied by allocating available water to these sectors. The focus of the current work is modeling global water system, its linkages with GGSM, modelling demand and computing stress. The specific sequence of calculations is as follows.

In order to elucidate the workings of the water system, a schematic diagram is incorporated (main manuscript line 182). The diagram and associated description are reproduced here:

> A schematic representation of the working of the water model of the GGSM is shown in Figure 1. On a global level, state variables are used with the sectoral intensities to obtain the total global sectoral demands. Geographical distributions for each of the sectors is used to disaggregate the total sectoral water demand into regional sectoral water demand. At this stage water demands for all the sectors and regions are available. Now, for each region all the sectoral demands are aggregated to get total regional water demand. Then, using the water availability for that region, regional water stress can be computed.

An example describing the model functioning is included in the main manuscript (line 190) and reproduced below:

> The working of the water model can be understood through the following example:

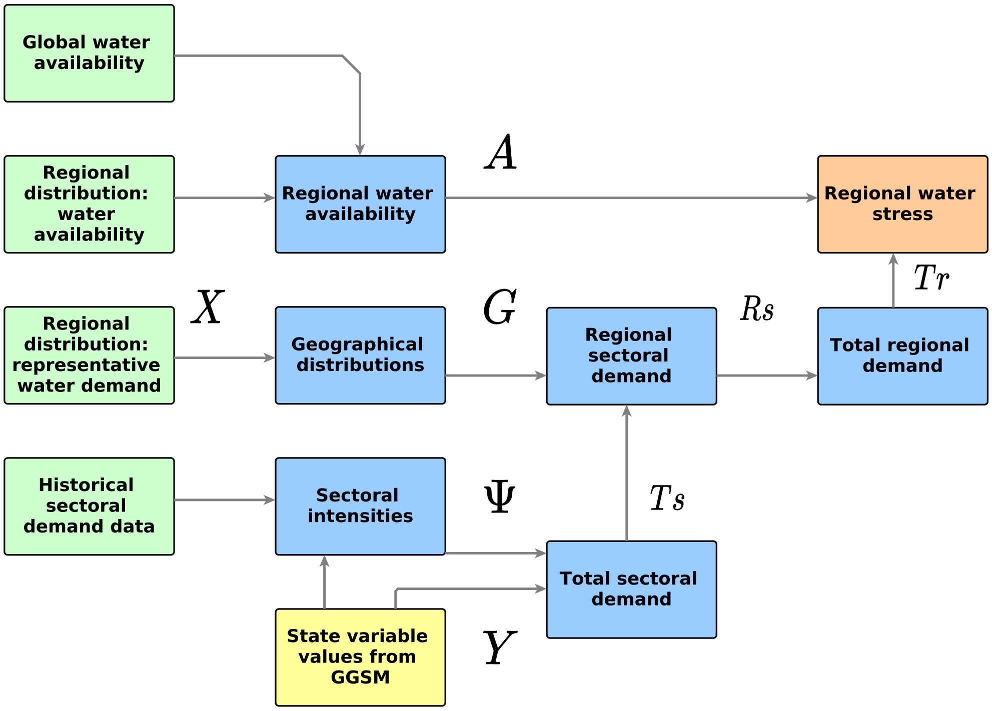

Fig 1. Schematic representation of water system modelling: Green colored boxes represent historical/literature based data, blue boxes represent interim variables, yellow box represents model outcomes based data and orange box shows the final outcome.

For the industrial sector, industrial production would be used to obtain the global industrial water demand. Using geographical distribution, regional IS demand is obtained. For a region, for example, Africa, total regional demand is computed from regional demands for agriculture, energy, municipal and industrial demands. This total regional demand is used to compute the water stress for Africa. In the context of supplying these demands, the water reservoirs which represent the stock of exploitable water are utilized. Here, specific modes of demand-supply are not considered, and the competition between sectors is ignored. A simple water balance based on material flow analysis is employed. The water stress is computed for all regions and sectors. If the water stress is $> 100\%$, it represents a situation where non-renewable water would be used.

Comment: How was the model validated (Figs. 2 and 3 are not clear at all)?

Response: We would like to respond to this comment by first explaining how the water demand is determined and then providing details regarding validation.

Sectoral intensity trends represent the transformation functions to obtain the water demand for a particular sector from corresponding state variables. The data source and the computation method along with the example of use of these trends is incorporated in the Model Parameterization and Validation section (line 211) and reproduced below:

> The sectoral intensity trends are used to compute the water demand for a sector using GGSM variable values. In other words, they represent the transformation functions to obtain the water demand for a particular sector from corresponding state variables. The historical sector-wise water demand data is obtained from the AQUASTAT database [8]. This country-level data is then aggregated continent-wise and mapped against state variable data (Y^i) for the same period to obtain the sectoral intensity plots (Ψ).

Now, the global sectoral demand model is validated by comparing the model outcomes (water demand for each of the sectors) with the historical data, as shown in Figure 4 (erstwhile Figure 3). The modified description of the validation (line 235) is reproduced below:

> Figure 4 shows the validation of the global sectoral water demand computation model. The markers indicate the historical global water demand for agriculture, industry, and municipal sectors. The historical data of industrial sector water demand also includes the energy sector. Accordingly, the model outcomes' global water demand of the energy and industrial sectors are aggregated before the comparison. The global water demand computed by the model for each sector, agriculture, municipal, and industrial (also including energy), conform well with the historical data.

Comment: What are the model limitations and assumptions?

Response: Critical assumptions, simplifications and limitations are listed below:

Assumptions

1. Seawater is ignored.
2. Only exploitable water, that is, the total surface water and regular renewable groundwater, is considered.
3. Fossil groundwater, desalinated water, environmental water requirements, and flows are not considered.
4. Effect of climate change and extreme weather events are not modeled in the present effort, but they could be addressed using this paradigm in future work.
5. Economics of water supply-demand is not considered.
6. Historical sectoral water intensity trends are assumed to be valid in future and the possibility of a disruptive technology becoming available is ignored.

Limitations

This work was subject to several assumptions and simplifications, which also form a basis for potential avenues for future research. These are as follows:

1. This work focuses only on the demand side. It does not delve into detailed modeling of the recycling of water which is a critical factor in the context of high water stress region. Moreover, often the water use efficiency improvement is at the expense of energy resources. Hence, the dynamics between water recycling in water-stressed regions and energy requirements can shed light on a trade-off between efficiency improvement and water recycling.
2. Secondly, the economics of water is not considered. This is a crucial limitation. Incorporation of water pricing will permit modeling the impact of stress on water demand, thereby providing greater insights.
3. The influence of climate change is another factor that is beyond the scope of the current work. Regional as well as global precipitation patterns can change because of climate change. Climate change may also influence the crop patterns, thus, altering the water intensity of the agricultural sector. Hence, one of the potential future research

avenues could be the modeling the climate change influences on water demand and availability.

4. Lastly, as a simplification, the regional aggregation of countries is carried out based on the continent in which that they are part of. However, a better approach would be grouping the countries based on the river basins they are sharing. Another simplification is aggregate modeling of the agricultural water demand. Incorporation of different crops and their water demand could make the model outcomes more realistic.

These are included in the Modelling global water system section (line 86) and conclusion section on (line 466).

Comment: Are the two-way interactions between food, water and energy taken into account? And if yes, how? I am sorry for the short review, but without these details, I am unable to provide any additional feedback on the results. At this stage, I thus recommend its rejection.

Response: Thank you for your valuable review comment. Two way interactions between different sectors mentioned by the reviewer are not taken into account in the present work. We are currently working on capturing these feedback effects and two way interactions. The scope of this work is limited to

- Modelling and integrating global water system with GGSM
- Capturing sectoral and regional demand dynamics
- Analysing the regional stress

Other significant changes

To address comments of Reviewer 3, model description equations have been revised and tables summarizing parameter values and variable description are included.

Reviewer 3

Comment: The authors present an interesting approach to model the water stress on the continents. However, there are still some parts that make it very hard to understand and therefore unsuitable for publication in its present form. The section Modeling global water system would improve a lot from a more detailed presentation of the equations and variables. Some problems currently are

Equation (1) uses $Q^i_{WR,s}$ which is not introduced at that point and uses indices i and s that are also not introduced.

Response: Thank you for your valuable review comment. Relevant indices are included (revised model description is included in response to next comment).

Comment: In equation (2) $Q^i_{WR,r}$ is introduced but it is a different variable than $Q^i_{WR,s}$. This is not recommended because it uses the same variable for two different flows.

Response: The variable description has been updated as per the reviewer's suggestion; and $Q^i_{WR,r}$, $Q^i_{WR,s}$ and $Q^i_{WR,\ s,\ r}$ are revised as Tr^i_r, Ts^i_s, and $Rs^i_{s,\ r}$, respectively. Additionally, equations for regional water availability and water stress computation are included for clarity. Relevant text and equations (line 132) are reproduced below:

> Equation 1 shows the utility of sectoral water intensity Ψ to obtain the total sectoral water demand Ts for sector s at timestep i from GGSM variables Y. Here, s is a sector in Agriculture ($P1$), Municipal (HH), Industry (IS), Energy (EP), or Livestock ($H1$).
>
> $$Ts^i_s = Y^i_s \times \Psi_s \tag{1}$$
>
> Equation 2 is used to compute the regional sectoral water demand Rs for region r and sector s.
>
> $$Rs^i_{s,\ r} = Ts^i_s \times G^i_{s,\ r} \tag{2}$$

Total regional water demand Tr can be computed by aggregating the regional sectoral water demand Rs for all sectors for a particular region as shown in Equation 3.

$$Tr_r^i = \sum_s Rs_{s,\ r}^i \tag{3}$$

To compute the regional water stress, regional water availability A is required. It can be computed using Equation 4.

$$A_r^i = M_{WR}^i \times Ga_r \tag{4}$$

where, M_{WR}^i represents global water availability at timestep i and Ga_r represents geographical distribution factor for availability for region r at timestep i

Now, using outcomes of Equations 3 and 4, regional water stress Ws can be computed using Equation 5.

$$Ws_r^i = \frac{Tr_r^i}{A_r^i} \times 100 \tag{5}$$

Comment: The variables in the text have a different font then in the equations (for example compare equation (3) with line 130 and 131)

Response: The model description is modified to ensure that variable text have same font in the equation and text. The same can be seen in the response of earlier comment.

Comment: Line 143: The meaning of variable X needs far more explanation. What is it representing? The same holds for X_r and X_{r_c}. I also recommend to always write into the sum over which indexes the sum is taken.

Response: Thank you for your comment. A more detailed description regarding variable X has been included in the revised manuscript. Further, the section describing

model has been modified to include an explicit and more readable (bulleted) description of the terms used in the equations.

The variable over which sum is taken are mentioned in the equations. However, since these variables are summed over a list of entities (for example, countries or sectors), instead of including an index, the set of all the corresponding entities is incorporated in the description of the equation.

Relevant text and equations (line 155) are reproduced below:

> The total value of the representative variable v for region r represented by X can be obtained using Equation 6a. Its distribution, that is, contribution of region r to the global value of sector s is represented by G. Here, v is a representative variable in Agricultural area, GDP, Meat production, or Population; c is a country in the list of countries in the world; and r is a region in Africa, Asia, Europe, North America, Oceania or South America. Equation 6b shows computation of G.

$$X^i_{v,\ r} = \sum_c X^i_{v,\ r,\ c} \tag{6a}$$

$$G^i_{s,\ r} = \frac{X^i_{v,\ r}}{\sum_r X^i_{v,\ r}} \tag{6b}$$

Comment: In equations (4) new variables X^i_r appear that are also not introduced.

Response: To address the comment, new variables appearing in the equations have been introduced. A detailed description of modified equations involving X has been included in the response of the earlier comment.

Comment: The readability would also improve significantly with the addition of a variable description table.

Response: We thank the reviewer for the valuable review comment. A variable description table is included and the same is reproduced here (Table 1):

Table 1. Variable description

GGSM variable/compartment	Symbol
Carnivores	C1, C2
Energy production	EP
Fuel source	FS
Herbivores	
Livestock	H1
Feral	H2, H3
Inaccessible resource pool	IRP
Industrial sector	IS
Inaccessible water reservoir	IWR
Primary producers	
Agriculture	P1
Grasslands	P2
Forests	P3
Resource pool	RP
Water reservoir	WR
**Water modelling variables**	
Availability of exploitable water (Regional)	A
Geographical distribution of water demand	G
Geographical distribution of water availability	Ga
Availability of exploitable water (Global)	M_{WR}
Regional Sectoral water demand	Rs
Total Regional water demand	Tr
Total Sectoral water demand	Ts
Water Stress	Ws
Representative variable	X

State variable		Y
Sectoral Intensity		Ψ
**Subscripts**		
Country		c
Region in	Africa, Asia,<br>Europe, North America,<br>Oceania, South America	r
Sector in	Agricultural, Livestock<br>Industrial, Energy<br>Municipal	s
Representative variable in	Agricultural area,<br>Meat production,<br>GDP,<br>Population	v
**Superscripts**		
Timestep		i

Comment: In the section Model parameterisation and validation

Maybe use M^i_{WR} instead of M_{WR} in line 167 because the value per week is given.

Response: Thank you for your valuable review comment. M_{WR} and M_{IWR} are are updated to M^i_{WR} and M^i_{IWR}. Relevant text (line 205) is reproduced below:

> Based on data from AQUASTAT [8], the total global exploitable water (M^i_{WR}) and the total renewable water resources are about 135 and 1060 billion cu m per week, respectively. These quantities are assumed to be constant over the simulation horizon for the current work. Inaccessible water is the difference between total renewable water and total exploitable water. Hence, inaccessible water resources (M^i_{IWR}) are 925 billion cu m per week.

Comment: I think the readability would also improve quite a bit if you also write the variable name for the parameters, as you did in line 167.

Response: To address the comment, the variable names are included in the parameterization section. Relevant portion of the revised text (line 212) is reproduced below:

> The sectoral intensity trends are used to compute the water demand for a sector using GGSM variable values. In other words, they represent the transformation functions to obtain the water demand for a particular sector from corresponding state variables. The historical sector-wise water demand data is obtained from the AQUASTAT database [8]. This country-level data is then aggregated continent-wise and mapped against state variable data (Y^i) for the same period to obtain the sectoral intensity plots (Ψ). Historical data is explicitly available for agricultural ($P1$), and municipal (HH) sectors; however, separate historical data for the industrial (IS) and energy (EP) sectors are not available as the water withdrawal data for the industrial sector also includes the energy sector. Hoekstra [6] analyzed the global water consumption by various sectors. From 1996 to 2005, they computed the combined industry and energy sector water demand to be 400 billion cu m per year. Mekonnen *et al.* [7] have computed annual global energy water for the period 2000-2005 to be around 250 billion cu m. Using this information, 62.5% of the aggregate industrial water demand can be allocated to the energy sector and 37.5% to the industrial sector. It is assumed that this allocation remains constant over the simulation horizon. The sectoral intensity for the livestock ($H1$) sector is linearly proportional to its mass.

Comment: The addition, a table showing each parameter a value and a source would also significantly improve readability for the readers.

Response: Thank you for the comment. Parameter value and source depicting tables are now included. Further, to improve readability, the section addressing

Table 2. Summary of model parameterization

**Parameter details**	**Values**	**Source**
Historical water demand and availability	M^i_{WR}: 135 billion cu m/week<br>M^i_{IWR}: 925 billion cu m/week<br>Agricultural, industrial and municipal water withdrawal	AQUASTAT [8]
State variable values	Mass of P1, H1;<br>Production of IS, EE;<br>Population	Nisal et al [10]
IS/EP split of industrial water demand	37.5%/62.5%	Hoestra [6]<br>Mekonnen et al [7]
Regional availability distribution	Africa: 9%,<br>Asia: 28.4%,<br>Europe: 15.2%,<br>North America: 17%,<br>Oceania: 2.1%, and<br>South America: 28.3%	FAO,<br>United Nations [5]

Table 3. Representative variables for water demand from different sectors

Sector	Representative variable	Source
Agriculture	Agricultural area	[11]
Livestock	Meat production	[12]
Industry and Energy	GDP	[13]
Municipal	Population	[14]

parameterization is split into two parts: first global sectoral demand parameterization and second regional sectoral demand parameterization. The parameterization summary is reproduced in Table 2 and Table 3:

Comment: In figure 1, there is no “EE” node but an “EP” node. Is this an error?

Response: Thank you for your valuable review comment. The text is corrected by replacing EE with EP.

Comment: The main function in the code currently has more than 2000 lines and therefore computations mentioned in the paper are hard to find. The code can be improved significantly if some parts inside the main function can be exported in different functions in other files.

I think the paper would also benefit from a further discussion of why the specific

> regions were chosen to reflect the continents. Large regions like Asia likely vary significantly between, e.g., West to East. It might therefore be necessary to further split such regions, while Oceania, for examply, seems rather insignificant compared to the other regions.

Response: We thank the reviewer for the valuable comment. As suggested demand computation has been taken out of the main code.

Selection of the regions based on continents was a decision governed by challenges of the data discrepancy, that arose out of changing boundaries of the nations within a particular region. As a consequence of selecting continents as regions the future projections are immune to boundary changes. Further, we have acknowledged the limitation pertaining to this simplification in the conclusion section. It is also an avenue of research planned to be carried out in future. The relevant text (line 483) is reproduced below:

> Lastly, as a simplification, the regional aggregation of countries is carried out based on the continent in which that they are part of. However, a better approach would be grouping the countries based on the river basins they are sharing. Another simplification is aggregate modeling of the agricultural water demand. Incorporation of different crops and their water demand could make the model outcomes more realistic.

References

1. IEA, Paris Global Energy Review 2021; 2021. https://www.iea.org/reports/global-energy-review-2021..

2. Rodell, Matthew, Isabella Velicogna, and James S. Famiglietti. Satellite-based estimates of groundwater depletion in India. Nature 460.7258 (2009): 999-1002.

3. United Nations, The United Nations World Water Development Report 2021: Valuing Water. UNESCO, Paris https://unesdoc.unesco.org/ark:/48223/pf0000375724.

4. Sadoff, Claudia W., Edoardo Borgomeo, and Stefan Uhlenbrook. Rethinking water for SDG 6. Nature Sustainability 3.5 (2020): 346-347.

5. FOOD AND AGRICULTURE ORGANIZATION OF THE UNITED NATIONS. Review of World Water Resources by Country; 2003. http://www.fao.org/3/Y4473E/y4473e0f.gif.

6. Hoekstra AY. The water footprint of industry. In: Assessing and measuring environmental impact and sustainability. Elsevier; 2015. p. 221–254.

7. Mekonnen MM, Gerbens-Leenes P, Hoekstra AY. The consumptive water footprint of electricity and heat: a global assessment. Environmental Science: Water Research & Technology. 2015;1(3):285–297.

8. Food and Agriculture Organization of the United Nations (FAO). AQUASTAT Database; 2021. http://www.fao.org/aquastat/statistics/query/index.html?lang=en.

9. Jekel CF, Venter G. pwlf: A Python Library for Fitting 1D Continuous Piecewise Linear Functions; 2019. Available from: https://github.com/cjekel/piecewise_linear_fit_py.

10. Nisal A, Diwekar U, Hanumante N, Shastri Y, Cabezas H, Boumans R. Integrated model for food-energy-water (FEW) nexus to study global sustainability: The main generalized global sustainability model (GGSM). submitted to PLoS ONE. 2021.

11. Ritchie H, Roser M. Land Use. Our World in Data. 2013;.

12. Ritchie H, Roser M. Meat and Dairy Production. Our World in Data. 2017;.

13. Roser M. Economic Growth. Our World in Data. 2013;.

14. Roser M, Ritchie H, Ortiz-Ospina E. World Population Growth. Our World in Data. 2013;.
