# Supplementary material for: Integrated model for Food-Energy-Water (FEW) nexus to study global sustainability: The water compartments and water stress analysis
Source: PLoS One. 2022 May 13;17(5):e0266554. doi: 10.1371/journal.pone.0266554 (PMC9106181; doi:10.1371/journal.pone.0266554)
Supplement: Attachment — Submitted filename: responsetoreviewers.pdf [file pone.0266554.s004.pdf]

Editor’s Comment

Comment: Thank you for submitting your manuscript to PLOS ONE. After careful consideration, we feel that it has merit but does not fully meet PLOS ONE’s publication criteria as it currently stands. Therefore, we invite you to submit a revised version of the manuscript. The reviewers now recommend to accept your work for publication, however, you have archived your code in GitHub. GitHub is not a suitable repository. GitHub itself instructs authors to use other alternatives for long-term archival and publishing, such as Zenodo. Therefore, please, publish your code in one of the appropriate repositories according to our code and data policy. In this way, you must include in a potential reviewed version of your manuscript the new repository and its DOI.

Response: We have put the code on Zenodo and new link is provided in the revised manuscript.
